# Lecture on Acute Rheumatism—Typhoid Fever—Pathological Anatomy of Phthisis

**Published:** 1840-02-29

**Authors:** W. W. Gerhard


### CLINICAL LECTURE.

### PHILADELPHIA HOSPITAL.

*Wednesday, February 19th, 1840.*

LECTURE ON ACUTE RHEUMATISM—TYPHOID FEVER—PATHOLOGICAL ANATOMY OF PHTHISIS.

By W. W. Gerhard, M. D.

*No. 14—Winter Course.*

To-day, gentlemen, I have an opportunity of exhibiting two very good examples of a disease which is very common towards the close of winter and during the spring, but of which no well marked case has as yet come under your observation. I mean acute articular rheumatism. But before I proceed to exhibit them, I wish to show you the progress of some cases of acute disease, which have already been before you.

The first patient is the man whom you saw last week, labouring under a slight attack of pneumonia. He is now perfectly recovered from the disease, and his case offers a good example of a very mild form of pneumonia, and of the readiness with which it yields to the simplest kind of treatment. The patient was taken ill on the 8th instant, and entered the hospital on the 11th; at that time he presented the signs of pneumonia, which I detailed to you at the last lecture. These signs continued in a greater or less degree until the fourteenth, from which time may be dated the period of his convalescence. The duration of this attack is, therefore, much shorter than that of ordinary cases of pneumonia, which, as I have already told you, usually extends to eighteen or twenty days, unless the treatment begin within the first three days. In a mild attack, like this, of course no active depletion was required. The treatment was commenced by the application of six cups to the right side of the chest, with the view of relieving the dyspnœa, and the congested state of the lung. The patient was put on an infusion of the asclepias tuberosa, as a diaphoretic, under the influence of which, the disease rapidly subsided. The case serves very well to illustrate the importance and propriety of a rule on which I have particularly insisted,—that we should never interfere, by active remedies, with a mild case of disease, which nature is evidently bringing to a favourable termination. Hence you will see that various modes of treatment receive a most unfounded credit, simply from the want of distinguishing between natural and therapeutic actions. The patient is still quite pale and emaciated; much more so than he ought to be after a trifling attack of simple pneumonia. From cases exhibited to you in previous lectures, you will doubtless be enabled to infer that the patient is threatened with phthisis. It may be laid down is a general rule, that in all cases of pleurisy and pneumonia, in which paleness and emaciation persist an unusually long time, the presence of tubercles, or at least a predisposition to their formation, may be suspected.

*Case* 2.—This is the dropsical patient whom you saw last week. The dropsy being of an acute kind, and depending on kidney diseases, a depletive and purgative treatment has been pursued from the commencement. A manifest improvement has taken place since you last saw the patient. The swelling in the upper extremities is almost gone; in the legs it is diminished; in the face it still persists almost as perceptibly as at first. The oppression of breathing, and all the other symptoms have declined in proportion. I shall continue the treatment, and hope for favourable results from it; still this variety of dropsy is exceedingly treacherous.

*Case* 3.—This is one of the examples of acute rheumatism. The attack is grafted on a chronic rheumatism; you saw the patient while the disease was in this state. On the 12th instant, it became acute; the patient suffered severe pain in the left shoulder and hip. The next day the pain and inflammation had extended to the same joints on the right side, and indeed, to almost every joint in the extremities, passing from one to another with great rapidity. The redness and swelling are at present most perceptible in the joints of the fingers; some days since they were well marked in the elbow and wrist, but since the knees and ankles became affected, the signs of inflammation at the elbow and wrist have evidently declined. The knee and ankle are now swollen, painful, and hot to the touch. The redness is not so manifest. This, in fact, is never so permanent as the other symptoms, and usually disappears altogether or in part, in two or three days: the tenderness, heat, and synovial swelling still remain, and may continue until the disease becomes chronic. On the other hand, rheumatism, when chronic, may become acute; it may continue for a long time, characterized only by pain and stiffness, but, at last, the particular epidemic tendency of the season, or some other cause equally efficient, increases the activity of the inflammation; then the joints become tense, red, and hot, and a very high degree of fever is developed. It was undoubtedly the character of the season, which brought about the change in the present case, as this is the period of the year when we usually begin to meet with acute rheumatism as a prevailing disease; it continues to prevail till the commencement of summer.

From the symptoms which I have enumerated, it appears that the affection in the present case is limited to the local inflammation, and the constitutional excitement, or fever. But in a large majority of cases, we meet with an important complication, viz., disease of the heart. Of this co-existent affection, we have an example in another patient of whom I shall presently speak. The disease of the heart consists in inflammation of its external and internal membranes, and the signs by which its existence is made known, are pain in the præcordial region, (which is absent in many cases, and rarely severe,) an irregular and confused action of the heart; the impulse, which is increased, unless there be effusion into the pericardium, by which it is necessarily diminished; dyspnœa; the bellows or rasping sound in the first sound of the heart; irregularity and weakness of the pulse, &c. But this affection may also be in a great measure latent throughout an attack of rheumatism, and may not become very obvious, until some time after the disease to which it owed its origin, had disappeared. Hence the great necessity of making a most careful examination of the condition of the heart, in all attacks of acute rheumatism.

*Case* 2.—Here is a woman who has also been suffering with acute rheumatism in several joints. But she has now nearly recovered from it; only a little pain and swelling exist at one of the wrists. In this patient, the disorder of the heart became manifest on the second day after her entrance into the hospital, or about a week after the commencement of the rheumatism, which is in entire accordance with the usual course of things. The signs of the cardiac disorder were very slight, as long as the rheumatism continued in active progress; since the decline of the latter, it has become more developed, and has been characterized by irregularity in the impulse of the heart, *bruit de soufflet* in the first sound of the heart, and dulness of the second sound. These signs are indicative of endocarditis. From this statement, you will be led to infer, that though the inflammatory affection of the heart may be so slight during the continuance of the rheumatism, as hardly to require any attention, it is apt to develope itself afterwards, and, if unchecked, to produce organic lesions of the heart. It is not the inflammation itself, therefore, that we have to fear, but its results.

The treatment of this case was commenced by bleeding, afterwards cups were applied near the upper part of the dorsal vertebræ. This treatment was soon followed by an alleviation of the pain and swelling, and a disposition to diaphoresis. The decoction of cimicifuga, or actæa racemosa, was next ordered with a view of promoting this tendency: it was first given to the amount of a quart, (containing two ounces of the root,) in the twenty-four hours, but as it produced some fulness and pain in the head, it was diminished to half the quantity. This article of late years has been highly recommended as a remedy in rheumatism, both acute and chronic. We have used the cimicifuga very freely in this house, in different forms. The tincture has been employed, in dozes of thirty drops to half a drachm or more several times a day; but even in these dozes we have found it very uncertain. The decoction, as above directed, has seemed more effectual. This remedy is said to have a valuable agency in alleviating the neuralgic pains which often accompany rheumatism, but this may be yet considered a matter of great doubt. There is no one disease in relation to which our positive knowledge of the precise effect of the different remedies is more deficient, than it is in inflammatory rheumatism. In the present case, it is impossible to tell whether the favourable issue is due to the cimicifuga, or to the general and local depletion. The latter have probably been the more efficient means in bringing about the result, for we know perfectly well, that when employed separately, bleeding and cupping to the spine or the inflamed part, are most useful means for reducing the general and local symptoms. Nevertheless, diaphoretics are very useful as adjuvants; since they promote that discharge which appears to be an ordinary and favourable symptom of the disease, and a suppression of which is invariably followed by bad consequences. If the cimicifuga has really the anodyne effect attributed to it, this would be an additional argument in favour of its use, because it is in every way a much safer remedy to employ with this view, than opium and its preparations.

To return to the first case of rheumatism which I brought forward: the treatment at first consisted entirely in the use of the cimicifuga; no depletion whatever was practised. But here the cimicifuga entirely failed, not the slightest improvement having followed its employment for a whole week; the quantity prescribed was an ounce of the root in decoction during the day. On the 15th, Dover's powder was substituted for it, and continued until yesterday. Leeches were then applied to the knee and ankle. The effect of all this treatment has merely been to diminish the pain somewhat in the elbow and wrist, and very decidedly in the knee after the leeching. This case would seem to be a fairer test of the powers of the cimicifuga than the other, because it was employed singly for several days. Not the slightest appreciable effect was produced by it; even the cerebral symptoms were absent.

The two preceding cases serve to illustrate the remarks which I made in a preceding lecture, as to the characters of acute rheumatism, its connexion with inflammation of the membranes of the heart, and the uncertainty of the remedies employed for its cure. Upon the whole, the best treatment appears to consist in general and local bleeding, conjoined with narcotics and sweating, unless the latter discharge should already be sufficiently copious; you know that it is one of the regular phenomena of the disease. The duration of inflammatory rheumatism is exceediugly various; it is sometimes checked in in its acute stage; frequently, on the other hand, it becomes chronic, and gives rise to other disorders.

Having spoken of the unpleasant effect upon the brain sometimes produced by the cimicifuga, it may be well to bring to your notice an instance of slight narcotism of a peculiar kind which lately occurred in our wards. A patient labouring under phthisis was ordered to take an expectorant mixture of syrup of senega, containing a small portion of the extract of hyoscyamus—a grain to the ounce of the mixture—so that about six grains of hyoscyamus were taken in the twenty-four hours. The mixture had an admirable effect in alleviating the cough; and no unpleasant effects were perceived until the night before last, (he had been taking the mixture about two days;) at that time the patient was perceived to be unusually drowsy; the face was flushed, and the pupils widely dilated. I found him in the same condition yesterday morning. This condition passed off by merely suspending the remedy. From what has been said, one might know at once that none of the disagreeable symptoms could have been produced by opium, but that they must have been the effects of some one of a particular class of narcotics, whose action is characterized by dilatation of the pupils. To this class belong belladonna, stramonium, hyoscyamus, and digitalis.

The narcotic effects of the hyoscyamus soon subsided after the expectorant mixture containing it had been withheld, and another substituted for it. This case ought to impress upon your minds the importance of attending closely to the operation of narcotic remedies, and of withholding them altogether, or reducing the dose, after they have been continued for a certain time. This precaution is often overlooked, and unexpected and anomalous modifications of the symptoms of a disease are frequently the consequence. The life of the patient may even be endangered by the uncontrolled operation of such remedies in the hands of an ignorant or inattentive practitioner. If the hyoscyamus had been continued for two days longer in the present case, there can be no doubt that its toxicological effects would have been developed. Digitalis requires even greater caution than hyoscyamus.

The next case is one which may be made to illustrate several points of pathology and therapeutics, which are of considerable interest. The patient entered the hospital on the 29th of January; he is by trade a shoemaker; is not intemperate, and has usually enjoyed good health. But on the 24th, after walking out in the cold, he was seized with chills, followed by soreness of the throat, pain in the right side of the chest, and difficulty of breathing. These symptoms continuing unabated, he was obliged, on the 26th, to quit his work. At the time of his entrance into the hospital, the following were the most important symptoms observed: slight pain in the right axilla; dulness on percussion; mucous rhonchus; respiration sighing; pulse weak and intermittent; face flushed. The weak pulse and sighing respiration indicated a peculiar disposition to sinking of the system. Sighing respiration, of itself, may be considered a conclusive evidence of this condition; and more particularly so, when it is accompanied by dulness and heaviness of mind, and a peculiar anxious or agonized expression of the features. When such a condition of things occurs, it is apt to produce a fatal result, unless closely watched and counteracted. This unfavourable tendency in such cases is the result partly of previous debility, and partly of the effect of the disease upon the nervous system; this effect being greatly disproportioned to the severity of the local affection. During the present course of lectures, I have insisted very particularly on the importance of attending to the organic lesions which constitute the essential features of diseases. But the functional disorders, both local and general, which result from such lesions, are no less important. This is proved by the present case, in which a structural change of trifling importance has given rise to a very serious functional disorder of the nervous system; that is, extreme despondency and feebleness. The same thing is noticed in malignant intermittents and remittents, in inflammation of the bowels, in diseases of the brain, and various other affections of frequent occurrence.

The great point to be attended to in the treatment of such cases is to counteract the depression by the milder diffusible stimulants. This patient has taken acetate of ammonia, and Hoffman's anodyne, with very good effects; a blister was also applied over the inflamed lung. Of course all active remedies for the reduction of the local inflammation, are out of the question. Sometimes even wine whey is necessary to keep the system from sinking; but usually, other diffusible stimuli are preferable.

Another instance of the same nature with the preceding has been offered by the next patient. He has had a slight attack of jaundice, and, during its course, took a mercurial purgative for its cure. The disease and the remedy together, produced a considerable degree of stupor and depression. The same thing is often observed in diseases of the kidneys.

At the lecture before the last, I told you that we had as yet seen no case of typhoid fever during the present season. During the succeeding week, however, a case presented itself, which I did not exhibit to you at our last meeting, because the symptoms were so slight as hardly to be recognised, unless by one accustomed to the observation of the disease. But I will mention the most important features of the case to-day, because it is probably the only opportunity which I shall have of calling your attention to the disease. This will enable me to complete the series of cursory remarks on fevers.

The patient is thirty-three years of age, a German by birth, and has lately emigrated to this country. I may remark, that it is among persons who have recently undergone some change of climate, and of their mode of living, that typhoid fever most frequently occurs; and in this country, it seems that Germans are more frequently the subjects of it than any other class of foreigners. Since he came to this country, the patient has been subject to chronic rheumatism. The present disease commenced ten days before his entrance into the hospital. The first symptoms were languor, costiveness, anorexia, &c., as in other fevers, followed by chills, and increase of the rheumatic pains. Typhoid fever often commences in an anomalous manner, so that it is difficult to tell at first what the disease is; among the anomalous symptoms is frequently observed, as in the present case, an aggravation of some pre-existing disease, if there be one. When the patient entered the hospital (on the 6th inst.) he was found to present the symptoms of typhoid fever, which I shall presently mention; but the case appeared so slight as to require no further treatment than dieting, and mild diaphoretics: the acetate of ammonia was prescribed. On the 10th, the patient was convalescent. The duration of the case was, therefore, much less than that of most cases of typhoid fever: the average duration being about twenty-one days.

The diagnosis was based principally upon the following symptoms: dizziness, partial deafness, somnolency, slight meteorism, tendency to diarrhœa, a rose-coloured, papular eruption on the abdomen, aud a few sudamina on the neck and chest. These symptoms are apt to be passed over without notice, when slight, as they were in the present instance, and no doubt are frequently thus overlooked by those who are not well acquainted with the characters of the fever. This disease, to which the name of typhoid fever has been applied, in order to distinguish it by a specific title, is in most instances sporadic; but in particular circumstances, it may become widely prevalent as an epidemic. I have already detailed most of the symptoms by which it is recognised; in addition to these, there are generally found more or less headach, oppression about the chest with mucous rhonchus, tenderness of the abdomen to pressure, and diarrhœa. In the case just detailed, there was no positive diarrhœa, but an extraordinary tendency to it: the patient was purged violently by a dose of castor oil.

The anatomical characters of typhoid fever consist of various lesions, some of which may be considered essential, and others simply accidental. Of the first class, the most important is inflammation of the glands of Peyer. This inflammation occurs in other diseases, but much less frequently than is generally supposed; and when it does occur, it is an entirely different affection from that which characterizes typhoid fever. This is a specific inflammation attacking the subjacent cellular tissue in its commencement: and it is met with in no other disease. Connected with this lesion of the glands of Peyer, there is in most cases, some alteration of the spleen, and absorbent glands of the mesentery; they are generally found enlarged and softened. The accidental lesions occurring in typhoid fever are extremely various. The inflammation may extend from the ileum to the stomach, and even to the pharynx; the brain and its membranes, as well as the thoracic viscera, may also be affected in various ways. The symptoms of typhoid fever, as well as the anatomical changes by which it is characterized, show it to be an entirely different disease from the typhus fever of the English. It is equally distinct from the sinking stage of remittent and other fevers, to which the term typhoid is frequently applied. Typhoid fever is not an uncommon disease in our own country. In New England, particularly, it frequently prevails. In the mountainous regions of the Middle states and Virginia, it is quite common, but in the malarious districts, it appears to be rather unusual. In the far South also, as I have been informed by gentlemen who have become familiar with the disease in this hospital, it is sometimes met with.

In connexion with this subject, it may be useful to make a few remarks in relation to the epidemic tendencies of different seasons, and the modifications thereby impressed upon the characters of prevailing diseases. It is in the winter that we most frequently meet with typhus and typhoid fevers, either in a sporadic, or epidemic form. But during the present season, we have not had a single case of the former, and but one of the latter. Such variations from the usual course of things are witnessed every year, and in relation to every form of disease. They are undoubtedly owing to some inappreciable modifications in the constitution of the atmosphere, which are dependent upon causes which it is impossible to ascertain. However this may be, the constantly varying characters of disease thus produced, demand the closest attention from the physician; for every such variation requires a corresponding modification of treatment: so that the same disease in different years, may call for the most opposite remedies for its cure. This subject received much more attention among some of the older writers, than it does at the present day. Sydenham, particularly, noticed the varying aspect of the epidemics which prevailed in London during his time; and he remarks that a physician should pay attention to the phenomena of the first few cases of every epidemic, and the effect of remedies upon them. From a few observations of this kind, he will be taught how to conduct himself through the whole course of the epidemic; for at each re-appearance of such a disease, it bears a certain physiognomy or general character, which attaches itself to nearly every case. Thus, there is sometimes an inflammatory, sometimes a typhoid tendency, and so on. This is true, not only of those diseases which are met with every where, but equally of those which are peculiar to certain localities or climates. These have certain general characters derived from the circumstances under which they occur, but constant as they are, they are subject to frequent modifications from the epidemic influences which may happen to prevail. This fact is familiar to every physician, who has had much experience in the miasmatic districts of the South. It is also illustrated by the history of the yellow fever of the West Indies. For many years this disease annually prevailed in the island of Martinique, and in a most destructive form. It then disappeared altogether for a considerable period; and when it again broke out, it was found to be greatly changed in its aspect. Instead of being uniformly fatal, it was so mild that less than five per cent. of the cases which were treated at the commencement, terminated unfavourably. It likewise attacked the old inhabitants, as well as strangers: whereas, in former years, it had been exclusively confined to those who were not acclimated. A corresponding change became necessary in the treatment of the fever. It is owing to annual changes of this sort, that remedies which are at one time looked upon as specifics for the cure of epidemic affections, at another time lose all their value, and unless the physician is prepared beforehand for such anomalies, he will often be disappointed and confounded, by the failure of plans of treatment which he had before believed to be infallible.

The remarks which I have made apply not only to those diseases which ordinarily prevail as epidemics, but to the most simple sporadic diseases. I have, during the present season, called your attention to this fact time after time, in speaking of pneumonia. This is one of the most strongly characterized of all affections,—so much so that it is often assumed as the type or standard of inflammatory diseases. Yet it is subject to perpetual variations in its character, as often as the season of its prevalence returns. This winter, as you know, it has been chiefly of an asthenic type, and in many cases it has even required a more or less stimulating treatment. In other years it is of the most violent inflammatory character, and demands the most vigorous depletion, and contra-stimulants. In the winter of 1834–5, when I was resident physician in the Pennsylvania Hospital, nearly all the cases of pneumonia were of the latter kind, and bleeding and tartarised antimony were as frequently employed as senega, and other stimulating expectorants are at present.

The principle which I have endeavoured to impress upon your minds, derives a further illustration from an epidemic which is at present quite prevalent in some districts of this city—I mean scarlatina. A great number of cases have occurred, but they are of a very trifling character, and require almost no treatment. I have met with no fatal cases of it, and it is probable that in cases which are treated from the beginning with remedies which do not interfere too much with the natural course of the disease, there is little danger. In general, nothing more has seemed to be required than to watch the course of the disease, assist the natural tendencies, and prevent the interference of injurious circumstances.

I have been induced to make the foregoing remarks by a conviction of the immense practical importance of the principle which they inculcate. My experience in this matter has been extensive, and I am fully persuaded of the truth of the maxim of Sydenham,—that an attentive observation of the peculiarities of the first few cases of each epidemic, will furnish the physician with a key to all the rest.

I shall conclude for the present by showing you a pathological specimen.

An old man entered the hospital on the 29th of January, in an advanced stage of phthisis. I examined him on the 30th, and found a strongly marked cavernous respiration under the right clavicle. The other symptoms were those of ordinary cases of phthisis, except that the dyspnœa was unusually great, and the respiration as rapid as thirty-six in the minute. These symptoms continued, with little variation, till the death of the patient, which took place yesterday. On opening the right lung, I find a large cavity at the summit, with hard and firm walls. It was this hardness of the walls of the cavity that gave rise to the extreme distinctness of the cavernous respiration, which was as loud and clear as the bronchial respiration of pneumonia. The remaining portions of the lung contain scattered tubercles in various stages of developement and softening, and the tissue around them is more or less congested. In the lower lobe especially, you observe a multitude of crude granulations of recent formation, around which the pulmonary tissue is gorged with blood, and partially hepatized. This is, therefore, an example of intercurrent pneumonia, which was here the consequence of engorgement of the lung produced by the severe dyspnœa which existed during life.

In the left lung also, there are numerous tubercles. At the summit we perceive the tissue of the lung contracted by the cicatrization of a former cavity. The history of the former attack of phthisis, which must evidently have occurred at some period or other of the patient's life, is involved in complete obscurity. The patient stated that he had never had any serious illness in his native country, (Ireland,) except a single attack of pleurisy; the phthisis, of which he died, did not commence until a long time after his arrival in this country.

The heart may be considered healthy. There is a very trifling diminution of the firmness of the muscular structure, a slight thickening of the mitral valve, and a minute deposition of cartilage at the bases of the semilunar valves of the aorta; but none of these alterations were of sufficient importance to have produced any disorder of the functions of the organ. The liver is fatty, and in a state of partial cirrhosis; the white granulations may be seen through its investing membrane.

